# Action of Purgatives

**Published:** 1878-10

**Authors:** 


Action of Purgatives.—The "Doctor" says ("Med. and Surg. Reporter") that Briger, experimenting by Moreau's method, finds that in all cases secretion is increased. There is not only transudation of fluid, but the contents showed the intestinal glands had acted freely. This applies to neutral salts and to drastics. The latter, in full doses, produce inflammation. All excite peristalsis. Simple laxatives, viz., senna, rhubarb, calomel, aloes, gamboge and castor oil, seemed only to excite peristalsis, without increasing secretion. The loops of intestine in which they were introduced by their active movements spread the drug all over the mucous membrane, and the aqueous part of an infusion of senna was removed.

